# The emergence of citizen collectives for care: the role of social cohesion

**DOI:** 10.1186/s12889-024-20780-7

**Published:** 2024-12-02

**Authors:** Kevin Wittenberg, Rense Corten, Tanja van der Lippe, Tine de Moor

**Affiliations:** 1 Utrecht University, Utrecht, The Netherlands; 2 Rotterdam School of Management, Erasmus University Rotterdam, Rotterdam, The Netherlands

**Keywords:** Collective action, Cooperatives, Social cohesion, Social identity, Social exchange, Citizen initiatives

## Abstract

**Background:**

Ageing populations and the ability to cure an increasing number of ailments put pressure on the health care sector. Meanwhile, care institutions retreat from rural areas and some governments emphasizes the need for citizens to find informal care primarily in their own social network. In The Netherlands, citizens increasingly respond by coming together to organize (in)formal care among themselves in ‘care collectives’. However, little is known about the conditions that need to be met for such collective action to develop, and explanations that go beyond an individualist perspective are particularly lacking. In this study, we aim to fill this gap, and specifically argue for the potential role of social cohesion to facilitate collective action among citizens through fostering a social identity, and through the prevalence of social relations that facilitate reciprocity and mutual trust among citizens. We further test whether these relations vary between municipalities, and whether they depend on the necessity for care services.

**Methods:**

We obtain data on the location of care collectives from an extensive Dutch inventory and match it to register data from Statistics Netherlands from 2020. We create measures for neighborhood attachment and contact using the ‘ecometric approach’. We test our hypotheses with multilevel logistic regression models and multilevel event history analysis for a subset of the data that can be analyzed longitudinally.

**Results:**

We find evidence for a positive association between neighborhood attachment and the emergence of a care collective, which is stronger if the necessity for care is higher. We do not find a relation between neighborhood contact and care collectives, nor do we find evidence that these relations vary between municipalities. We cannot replicate our positive associations in the longitudinal model, and thus remain reserved about their causal interpretation.

**Conclusions:**

There is considerable variability in the extent to which neighborhoods organize care services collectively. Partly, this may be attributable to differences in the prevalence of neighborhood identity, which would imply that an increasing reliance on citizen collectives may increase inequality in access to healthcare. Further research should emphasize combining community-level information with data on individual participation in care collectives to delve deeper into the dynamics of invitation, representation and embeddedness than current data allows.

**Supplementary Information:**

The online version contains supplementary material available at 10.1186/s12889-024-20780-7.

## Background

Ageing populations [1], the need for budget cuts [2], privatization [3] and the ability to cure an increasing number of ailments are among factors that have resulted in a rising pressure on the health care sector. As care institutions withdraw from rural areas, and governments emphasize the need for citizens to find informal care first and foremost in their personal network [4], citizens increasingly come together to arrange care services themselves. To do so, some organize themselves in citizen collectives, defined as institutions that are started, maintained and owned by citizens, with the aim to set up a provision of services for their members for an extended period of time [5, 6]. Some of these services are informal, such as a grocery service, DIY service or shared dining initiative. However, collectives can also arrange formal services for their members, such as the collective buy-in of weekly physician consultation, or the construction of sheltered housing units. The emergence of these citizen collectives for care is part of a larger movement of rising collective action of citizens, spanning across multiple domains (e.g. housing [7], insurance [8], renewable energy provision [9] and retail [10].

While citizen initiatives in these other domains have become more common, citizen collectives for care still appear to be relatively scarce, and only recently gained traction in some countries, such as Italy [11], the UK [12] and the Netherlands. The Netherlands in particular can be seen as a testing ground for these collective approaches to care, as its collectives have not only experienced a period of rapid growth, but they have also organized themselves in overarching networks to increase their coordination, knowledge sharing and legitimacy as institutional partners [13, 14].

Because care collectives are local, often informally organized phenomena, an empirical assessment of their prevalence has remained absent until recent research has exploited the centralizing efforts of the Dutch network of care collectives to map these care collectives in The Netherlands [15, 16]. This research showed that the number of citizen collectives has been growing at an increasing rate for the past two decades [16]. However, more importantly, it also exposes that this growth of collectives is strongly unevenly distributed across the country [15]. The collectives appear in distinct clusters and are particularly present around the southeastern and eastern rural areas of the country, as well as some of the major cities. This raises the question why citizen collectives emerged in some areas of the Netherlands, but not in others. Since the Netherlands is spearheading an expansion of citizen collective action into the care domain, insights into the conditions for their emergence can provide fruitful insights for communities and lawmakers in-and outside of the Netherlands.

Previous research on the emergence of care collectives has identified various individual motives to join a care collective, as reported by current members [17–20]. These studies highlight the importance of both social and personal motivation. Social motivation refers to the feeling that citizens want to improve facilities for their neighborhood, and share a feeling of collective responsibility [21]. Personal motivation refers both to the notion that being a part of the collective brings citizens ‘fun’ social contact, as well as the prospect of future returns, where members hope to use the services of the collective themselves in the future [22]. Next to these motivations, studies indicate that the (perceived) capacity to take collective action, and direct ties to people that were already in the collective were causes of membership [18].

However, there are three major reasons why these findings are insufficient to explain the geographic variation in care collectives. First, the motives to join care collectives that have been uncovered in previous research have almost exclusively been studied from an individualist perspective. Hereby, these motives were either treated as given, or they were explained from individual attributes of the members. However, for the geographical pattern of care collectives to arise solely from these individual traits, the socio-demographic segregation of the Netherlands would have to be far stronger than it is. This means that additional explanations for the emergence of collectives, situated at the community level, need to be considered.

Second, research on care collectives to date is almost exclusively qualitative, based on interviews with key stakeholders of existing collectives. While these interviews provide valuable insights into the processes that guide the development of the collectives that were studied, the results are sensitive to potential survivor bias. That is, they only investigated the processes that govern the emergence of a citizen collective that successfully emerged. These insights are not sufficient to explain why care collectives did *not* emerge in other areas.

Third, research to date has focused on motivations of the very first initiators of care collectives, who were involved in setting up contact with municipalities and mobilizing their neighborhood. However, for a care collective to materialize, a larger group of citizens that chooses to join the collective needs to be considered. Their reasons for joining the collective may well differ from those of the first initiators. Research that considers motivations of this larger group of citizens is scarce and non-peer-reviewed (see [18, 20, 22] for insights from professional literature).

To our knowledge, there has been only one study [15] that has considered the role of community-level characteristics in the emergence of care collectives. While this study had not developed a theoretical framework from which explicit mechanisms could be derived, initial results hinted at the importance of a neighborhood’s capacity for collective action, and a potential role for a neighborhood’s social capital to facilitate this capacity.

In the current study, we further this strand of research. We focus on the level local neighborhoods, and we argue that the mobilization of citizens is an important aspect of a neighborhood’s capacity to engage in the collective action required to start a care collective. We conceptualize van der Knaap et. al.’s notion of social capital as social cohesion, and argue that cohesion is one of the main factors that can influence the mobilization of citizens to start a care collective. Specifically, we hypothesize that social cohesion can foster collective action of citizens through the establishment of a collective identity and through the establishment of social control and norms of cooperation and reciprocity, and substantiate these arguments theoretically by drawing from literature on social identity and social exchange.

Taken together, we aim to answer the following research question: To what extent and under which conditions does the social cohesion of a neighborhood facilitate the emergence of a care collective? To address this question, we obtain an updated and more extensive version of the inventory of citizen care collectives that was used in previous research [15] from the Dutch network of care collectives ‘Nederland Zorgt Voor Elkaar’ (‘*Netherlands cares for each other’*). The locational data of these collectives is matched to information about social cohesion from the Dutch Housing and Living survey (WoON) and to public register data from Statistics Netherlands.

We identify four key contributions of our study to the literature on citizen collective action. First, we investigate how communities differ in their resilience to a retreat of care institutions. In doing so, we go beyond previous research by investigating causes for the emergence of citizen collectives at the neighborhood level. While previous research has argued for the importance of a mobilized neighborhood and mentioned the potential importance of social relations from an individualist perspective [21, 22], we explicate how social cohesion may be the relevant concept at the community level that captures these social effects and test these expectations quantitatively. For a subset of the care collectives, we have information about the year they were founded. We leverage this information to strengthen the causal claims of our hypotheses.

Second, our analysis includes neighborhoods where a citizen collective emerged, but also cases where it did not. As such, we address the fact that previous research may have been subject to survivor bias. Third, we account for the interaction between social cohesion and other theorized causes of care collectives at the neighborhood level: the necessity for a collective and the availability/legitimacy of a model of collective action [6, 15, 17].

Fourth, this research contributes to a broader societal discussion around care collectives. Since care collectives are able to provide highly customized local care solutions [23], and reduce the workload of care professionals and informal caregivers [24], they have been proposed as central institutions in an alternative approach to care. However, the emergence of collectives requires collective action by citizens, and is not self-evident. The results of this study contribute to informed governmental interventions aimed at promoting the emergence of care collectives, and helps to identify areas where citizens are less resilient to the retreat of care institutions. This can help to understand how an increasing importance of care collectives in the care domain can result in regional inequality with respect to access to health care.

### Theoretical background

The purpose of a care collective is to provide care services to citizens where the market and government have failed to meet demand or quality standards for these services [25, 26] (see [27] for a historical perspective in relation to their current re-emergence). It can do so by providing a platform for informal care between neighbors, but it can also serve as an institution through which citizens exert influence on local governments and organize formal care collectively. Importantly, while citizen collectives can provide informal care internally between members (e.g. grocery shopping and cooking by volunteers for the elderly members), they do not typically provide professional healthcare themselves. Instead, citizen collectives may purchase private care collectively, or constitute a conversation partner for the municipality to allocate existing funds more efficiently and targeted to the needs of their members. However, before collectives can fulfill these roles, their emergence usually passes through several stages [18] (see [28] for a list of recent examples). First, an initiator or group of initiators determine the demand for care in a neighborhood that is not met by any institutions that are currently active in the area. Often, the initiators (also referred to as primary movers) are active citizens with a background in the care domain, but there are also instances where the municipality takes this initiating role [18].

The initiators assess the possibilities for funding, partnerships and the initial services that the collective should provide. After they have done so, the collective can either take a formal judicial structure, or remain informal. The next step for a collective is to attract members, volunteers and/or participants [18, 29]. In this stage, a larger group of citizens needs to be mobilized to join the collective. This initial mass of citizens is essential to fill voluntary positions, obtain financial means to organize formal services and to ensure that the collective offers sufficiently tailored services.

The early members of a collective will play an important role in taking on some of the voluntary positions in the collective, as well as making decisions about the course of the collective. At the same time, an emerging collective can offer fewer formal and informal services than a collective that is already well-established, and is still in an (economically) fragile position. As such, the behavior of joining a collective at this stage is seen as costly. This means that the mobilization of citizens for an emerging collective can be seen as a problem of collective action [30], where the contributions of citizens are long-term investments in the provision of a public service for the neighborhood.

In the sections below, we pay attention to the concept of social cohesion and argue for two ways in which it can affect the willingness of citizens to engage in such costly behavior. We argue that these mechanisms are driven by two different aspects of social cohesion, a sense of neighborhood attachment and the prevalence of social relations, and derive hypotheses to test these mechanisms. Finally, we derive expectations about the interplay between social cohesion and other theorized causes of care collectives: The demand for a care collective and the presence of other collectives in the municipality.

There is a fragmented view in the literature on how to define social cohesion. Since it is the central explanatory concept in this study, it is important to determine what we consider social cohesion to be. Common to many contemporary definitions of social cohesion is a complex interplay of factors at the individual, institutional and community level that pertain to the well-being, belonging, connectedness and inclusivity of group members (see [31] for a review). We follow this conceptualization here, and argue that two aspects of cohesion are of particular importance when we consider the emergence of a care collective. First, we argue that the structure of social relations is an important aspect of cohesion, because it can influence the establishment of trust and reciprocity through social control. Second, we argue that the feelings of belonging and attachment in a neighborhood are a relevant aspect of cohesion, because this explains how altruistic behavior towards other members of the neighborhood can emerge as a result of neighborhood identification. These two mechanisms also relate closely to the self-reported ‘personal’ and ‘social’ motivations that were reported in explorative qualitative research [22].

### Belonging and attachment: Neighborhood identification

The first mechanism through which neighborhood cohesion can facilitate the emergence of citizen collectives builds on a feeling of belonging to the neighborhood and is rooted in the social identity literature. A central element of a cohesive neighborhood, is a sense of attachment and belonging [32]. These feelings reflect that citizens consider their membership of the neighborhood an important part of their social identity [33]. Attachment and belonging to the neighborhood have been found to result from various sources, such as everyday interactions in the same local setting, or perceived (demographic) similarity to other inhabitants [34].

If citizens feel that the neighborhood in which they live is an important part of their identity, this can cause them to consider collective goals of the neighborhood as their own goals [35, 36]. In the case of care, this means that citizens who identify strongly with a neighborhood, value a proper provision of care for their entire neighborhood, even if they do not require such services themselves. As an example, they may internalize the goal that the elderly in the neighborhood, who are required to move away to receive the care they seek, should be able to receive this care closer to home.

Subsequently, the value that inhabitants attach to these neighborhood-level goals can motivate them to contribute to the collective action that is required to achieve them. Thus, citizens who value the provision of formal and informal care in the neighborhood are more likely to contribute to the establishment of a care collective than citizens that do not value such goals. The neighborhood identity plays an important role in this process, because it marks the boundary of who does and does not belong to the group that one is willing to provide care for [37]. Thus, we expect that neighborhoods in which the average feeling of attachment and belonging is higher, are those in which a larger number of inhabitants is willing to contribute to the establishment of a citizen collective. Thus, a collective is more likely to emerge in those neighborhoods.

In summary, a cohesive neighborhood can foster a strong social identity among its inhabitants through their feelings of attachment and belonging. This identity motivates people to value and contribute to the collective provision of care for the neighborhood, which makes the emergence of a citizen collective more likely. We derive the following hypothesis:

H1: There is a positive association between the average feelings of attachment and belonging in a neighborhood and the probability that a citizen care collective emerges in that neighborhood.

### Social relations: reciprocity and future benefits

While individuals in a cohesive neighborhood may be mobilized for collective action by their sense of attachment and belonging to the neighborhood, and thus by regard for the goals of others, they may also be driven by prospective *personal* benefits. For instance, citizens may wish to use the services of a citizen collective themselves in the future. Additionally, citizens may contribute to a collective to establish a positive reputation within the neighborhood, aiming to establish or maintain a reciprocal relationship with other citizens for the exchange of social and material resources.

For any given citizen, the extent to which their contribution to a collective translates into future benefits is uncertain for two main reasons. First, it is uncertain whether the citizen will acquire a return on their contribution in the future. For instance, consider the case that a citizen aims to use future services of the care collective themselves. If said citizen moves to another city before they require any of the collective’s resources, or if they remain highly vital until their death bed, they may never need to appeal to the collective. The second source of uncertainty stems from the fact that the future benefits that a citizen can obtain from a contribution to the collective also depends on the current and future actions of other citizens. While neighborhood cohesion is less straightforwardly connected to the first source uncertainty, we argue that it is especially likely to reduce uncertainty associated with the dependence on actions by others. Particularly, the prevalence of informal social relations between citizens is expected to facilitate trust and reciprocity and increase interactions’ *‘shadow of the future’* [38].

Building on social exchange literature (see [39] for a review), we argue that the prevalence of informal social relations in the neighborhood (e.g. the prevalence of conversations, communal barbeques etc. among inhabitants) can foster cooperative behavior among citizens that are driven by personal benefits both directly and indirectly.

Directly, a tightly-knit neighborhood facilitates rapid and widespread diffusion of information across the neighborhood. This facilitates a high level of social control [40, 41], which refers to the sanctions or rewards one may incur based on their behavior, (i.e. social or material resources that are granted or withheld by other citizens in the neighborhood) [40].

Since information about the previous behavior of a citizen (i.e. their positive or negative reputation) can spread more rapidly and more widely in cohesive than in sparsely connected neighborhoods, the magnitude of these future sanctions and rewards is higher in cohesive neighborhoods. As the future benefits of contributing to a citizen collective grow, so does the likelihood that one will contribute to the establishment of a collective. Empirical research finds some support that the density of social relations indeed fosters cooperative behavior in other settings (e.g [42]). To translate these findings to our research, we assume that contributing to a care collective in the neighborhood is seen as pro-social behavior by others in the neighborhood. Thus, taken together, we argue that citizens who are motivated for cooperation by the fact that they will be indirectly reciprocated (i.e. rewarded) for this behavior will be more likely to contribute to a care collective in a cohesive neighborhoods.

Indirectly, a community characterized by tightly-knit social relations can affect the uncertainty citizens feel about the extent to which they can use the services of a collective in the future. Here, social control is unlikely to exert direct influence on one’s future ability to use the collective’s resources. Exclusion from health care collectives would be a strong sanction for the absence of contribution to its emergence, and is in direct contrast to the core value of openness and accessibility that is generally maintained by collectives [43]. While collectives do sometimes limit the opportunity for membership to residents of a certain neighborhood, they are generally considered non-excludable to residents of that neighborhood. However, while we do not expect sanctioning behavior in this context, an extended period of social control, cooperative relations and social learning can still contribute to the establishment of norms of reciprocity and trust in a neighborhood over time [44].

In a neighborhood that is characterized by such norms, citizens can be more certain that current and future residents will continue to adhere to this norm, and thus behave comparably cooperatively. In our case, that cooperative behavior refers to the provision of required contributions to the citizen collective to ensure its stability in the future. Thus, citizens that are motivated by the potential future use of a care collective’s resources are also more likely to invest in the establishment of such a collective in socially connected neighborhoods.

Taken together, the informal social relations between citizens in a neighborhood can foster cooperation among citizens directly through social control and indirectly through the emergence of norms of reciprocity and trust. As a result, a larger number of people are likely to be willing to invest in the establishment of a care collective in neighborhoods with many informal relations, and the likelihood that a care collective emerges is thus higher in these neighborhoods. This leads to the following hypothesis.

H2: There is a positive association between the prevalence of informal social relations in a neighborhood and the probability that a citizen care collective emerges in that neighborhood.

### Interaction between cohesion and other predictors

Previous research has pointed at two features at the community level, other than social cohesion, that may be important for the emergence of care collectives: The demand for a care collective and the capacity of citizens to navigate the municipal bureaucratic and judicial environment. Indeed, both qualitative [17] and quantitative [15] inquiries have found evidence hinting at the importance of these factors. While research to date has studied these effects in isolation, we pose that these two neighborhood characteristics may interact with the mechanisms that are driven by social cohesion.

First, the demand for a care collective in a neighborhood is expected to exert a direct influence on the probability that a care collective emerges in that neighborhood. That is, in neighborhoods where there aren’t many elderly, or established care services are very well equipped to deal with the care demand of the neighborhood, the emergence of a care collective is less likely than if the demand for the services of a care collective is higher.

We follow previous research in measuring the demand for a care collective by the number of elderly residents in a neighborhood, defined as the number of residents that is 65 years of age or older [15]. This is supported by the fact that most care collectives in our inventory indicate that fulfilling the needs of elderly citizens is among their main foci. This measure of necessity is quite broad, but an important benefit is that it is well-documented and available longitudinally for most neighborhoods of the Netherlands. We discuss alternative operationalizations and robustness in the results section. Thus, we derive the following hypothesis.

H3a: There is a positive association between the number of people that is 65 years or older in a neighborhood and the probability that a citizen care collective is established in that neighborhood.

In addition, we expect that there is an interaction between the effect of social cohesion and the necessity for a care collective in a neighborhood. That is, if there is no demand for the services of a care collective, the effect of neighborhood cohesion on the emergence of a care collective is expected to be weaker. This is because care collectives (usually) emerge in response to a mismatch between care supply and demand in the neighborhood [45]. If the demand for care services is low, such a situation is less likely to occur. Conversely, the effect of care demand on the emergence of a care collective is also expected to be higher if the social cohesion is higher. This, we argue, is because social cohesion facilitates the mobilization of citizens that is required for the collective action of starting a care collective. In addition, the care demands are likely to be more visible and communicated between citizens of the social cohesion is higher, which increases visibility of care demands in the neighborhood. Taken together, we derive the following hypothesis.

H3b: The association between feelings of attachment and belonging in a neighborhood and the emergence of a care collective in that neighborhood is more positive if the number of people that is 65 years or older in a neighborhood is higher.

H3c: The association between the prevalence of social relations in a neighborhood and the emergence of a care collective in that neighborhood is more positive if the number of people that is 65 years or older in a neighborhood is higher.

A similar argument can be made for the capacity of neighborhoods to navigate the judicial and bureaucratic environment when starting a collective. Citizens that aim to start a collective, often visit existing care collectives to develop a similar course of action and imitate existing organizational structures [46]. This ability to imitate and learn from established collectives enhances a community’s capacity for self-organization [15]. Previous research has captured this effect by analyzing the linear distance from a care collective to other collectives [15]. However, it is difficult to interpret such a relation without considering other explanations, rooted in culture or local policy, that could explain geographic clustering of collectives.

Instead, we consider the presence of other citizen collectives in the same municipality as a conceptualization for the capacity for neighborhoods to navigate judicial and bureaucratic processes in self-organization. The reasons for this are threefold. First, this concept still captures whether citizen collectives emerge in close proximity to other citizen collectives, but it additionally captures a meaningful shared (policy) context of the collectives. Second, it reflects the extent to which municipalities are familiar with collective institutions of citizens, which increases their legitimacy, and is an indicator for the extent to which municipalities are supportive of self-organization by citizens. Finally, the measure is less susceptible to care collectives that are outliers in terms of distance to other collectives.

Social cohesion is expected to facilitate the emergence of care collectives especially in neighborhoods that are part of a municipality that already contains other care collectives. As argued before, neighborhoods that are located in a municipality where other collectives already exist, have more opportunities to learn from each other about which practices are efficient, and are more likely to receive cooperation from local government. This leads to the following hypotheses:

H4a: There is a positive association between the existence of another care collective in the municipality that a neighborhood is a part of and the probability that a citizen care collective is established in that neighborhood.

H4b: The association between feelings of attachment and belonging in a neighborhood and the emergence of a care collective in that neighborhood is more positive if another care collective exists in the municipality of the neighborhood.

H4c: The association between the prevalence of social relations in a neighborhood and the emergence of a care collective in that neighborhood is more positive if another care collective exists in the municipality of the neighborhood.

## Methods

### Data

To test our hypotheses, we require data on the neighborhoods in which care collectives have been established. An inventory of 1482 care collectives and the neighborhoods in which they are located is obtained from Nederland Zorgt voor Elkaar (NLZVE), a network and interest group of Dutch care collectives. This inventory was constructed by NLZVE from 2 sources. The first is a list of self-reported care collectives that registered on the website of NLZVE. The second is an inquiry among the informal network of care professionals, legislators and members of local networks of care collectives that were known to NLZVE. The snapshot of this inventory that we have obtained contains all care collectives that were registered before April 27th, 2021.

All initiatives that are registered in this inventory meet a number of criteria. First, they are citizen-initiated and citizens have (shared) ownership of the initiative (citizen-owned). Second, the initiatives focus on improving the local living environment of its participants (neighborhood-oriented). Third, the initiatives strive for the long-term production of goods or services, rather than one-off or temporary events (long-term). Fourth, the initiatives are related to the domain of (in)formal care and welfare (care-oriented). Finally, the initiatives are open and accessible to everyone in their target audience that wishes to participate (inclusive character).

Information about 323 care collectives in our inventory is enriched with the year in which these collectives were founded. We obtain these data from a survey among care collectives, conducted by NLZVE in collaboration with Dutch research institutes Vilans and Movisie in 2019. The survey was set out among the inventory of NLZVE and was publicly available on the websites of NLZVE, Vilans and Movisie from September 15 to November 20 2020.

Next to the locations of care collectives, we require a measure of contact and of neighborhood attachment in Dutch neighborhoods. These attributes are constructed using the Dutch Housing and Living Survey (WoON), which was conducted trilaterally from 2006 to 2018 among a stratified sample (resampled for each wave) of inhabitants of Dutch neighborhoods [47]. The number of respondents varies between 50,000 and 70,000 over the years.

The number of elderly residents in each neighborhood is obtained from public register data. We also obtain various control variables from public register data, aggregated to the neighborhood level by Statistics Netherlands (CBS).

### Operationalization

For the analyses, few control variables are available before 2014. To that end, we perform a cross-sectional analysis in which we include all relevant control variables as well as a longitudinal analysis without controls. For this reason, all operationalizations include a cross-sectional and a time-varying version.

### Dependent variable: Care collectives

We use the locational information of the care collectives in our inventory to determine in which neighborhoods a care collective has emerged. The ideal geographic level at which to study the mechanisms, is the level at which people form communities. What we have thus far called ‘neighborhoods’, we define as four-digit zipcode areas. The advantages of this operationalization are threefold. First, it is in line with previous research [15, 48], which increases the comparability of our results. Second, the area in which people are expected to know one another, and the area which is deemed most relevant for inhabitants’ social identification, likely differs between urban and rural areas. In rural areas, these processes are often investigated at the level of an entire village [49, 50], while in urban settings, they are often associated with the level of city districts [51, 52]. Four-digit zipcode areas reflect this difference by scaling in surface area based on the number of people that reside in an area. Thus, in sparsely populated rural areas, zipcode areas cover entire villages and surrounding houses, while city zipcodes relate more closely to city districts. Finally, the data we have obtained are limited to this level of analysis. Further reduction in neighborhood size is possible for some regions, but much information would be lost to prevent violation of respondents’ privacy. The median number of inhabitants for a zipcode area is 2590.

We identify zipcode areas in which a care collective has emerged through a dummy variable, taking on a value of unity if at least one care collective is recorded in that area. We do not distinguish between additional care collectives in the same zipcode, since we have no additional information about these collectives, such as the number of people that are involved in each collective or the exact services each of them offers. This makes it difficult to interpret the existence of multiple collectives. In total, we find that 896 out of 4069 Dutch zipcode areas have at least 1 established care collective, and 318 of these zipcode areas have more than 1 (Median = 2, IQR = 1) recorded collective. We use this dummy variable in the cross-sectional analyses.

In addition, we create a zipcode-period file of all the zipcode areas from 2006 up to 2018. We keep all zipcode-year combinations for those zipcodes in which a care collective has emerged of which we know the year in which it was founded. We exclude all zipcodes of which we know a collective has emerged, but we do not know in what year. That is, we exclude all zipcodes that occur in the total inventory of NLZVE, but not in the subset which filled out the survey. We are thus left with zipcodes of which we know when a collective was founded, and zipcodes for which we are relatively certain that a care collective has not emerged yet. For each zipcode-period observation, a dummy variable denotes whether a care collective was founded. We use this variable for the longitudinal analysis.

### Independent variable: Social cohesion

The two dimensions of social cohesion we consider are *attachment* and *contact* in the neighborhood. Contact in the neighborhood is measured by three statements pertaining to the respondents’ personal relations to direct neighbors and others in the neighborhood, as well as the respondents’ perception of general interactions in the neighborhood (see Appendix A for an overview of all items). Neighborhood attachment is measured with the statements: “I am attached to this neighborhood” and “I feel at home in this neighborhood”. For all of the above statements, responses were recorded on a five-point Likert scale, ranging from “Completely agree” to “Completely disagree” (items are recoded such that higher values indicate a higher score of social cohesion). Item non-response was singularly imputed by Statistics Netherlands through predictive mean matching.

We summarize and aggregate these items to the neighborhood level using an ecometric approach [53–55], in line with previous research [15, 48]. This approach aims to capture variance in social cohesion that is inherent to the neighborhoods, net of variation between individuals. This comes down to fitting a multilevel item response model for each of the constructs, in which we include a number of respondent characteristics. The neighborhood-level residuals of this model then reflect the deviations of contact and attachment in a particular neighborhood from the general average.

The advantages of an ecometric approach over other methods of aggregation, such as calculating the mean or standard deviation of the survey items per neighborhood, are threefold. First, an ecometric approach accounts for the fact that respondents’ perception may be influenced by personal characteristics. For example, perceptions of contact in the neighborhood may be systematically lower for older respondents, since they compare the contact to the past, whereas young respondents do not. Second, it accounts for the fact that neighborhoods vary in the number of survey respondents, by shrinking deviating neighborhoods with few respondents to the general average [56]. Third, it accounts for the interdependence between the different items that measure each dimension of social cohesion through a multilevel structure. For a more detailed description of the approach, see Appendix B.

For our application of the ecometric approach, we account for 8 characteristics at the respondent-level. Of these characteristics, age, gender, education, years of residence, income and whether the respondent bought or rents their home, have been included in accordance with previous research [15, 48]. In addition, we add information about whether the respondent has children living at home and whether the respondent has a migration background (distinguishing between first and second generation). We include these variables because they likely relate to (perceptions of) neighborhood contact and attachment. For example, parents of non-adult children more often have contact with neighbors and are the target audience for many neighborhood events [57]. For migration background, inhabitants with a migration background more often experience language barriers and have different perceptions about neighborhood attachment than inhabitants without a migration background [58]. An overview of all subject-level variables and their operationalization can be found in Appendix A.

In total, we fit 10 response models. One for neighborhood contact and one for neighborhood attachment per year of the WoON survey. We thus obtain measures for 2006, 2009, 2012, 2015 and 2018 (Appendix B). The values for intermediate years are linearly interpolated. For the cross-sectional model, we use the values of attachment and contact from 2014. We choose this year because it is the year of the WoON survey that was just before the majority of care collectives were founded, based on the subset of the data for which we know the year of emergence. In addition, it is the first year in which all the relevant control variables are available. By choosing this year, we hope to strike a good balance between preventing conclusions based on reverse causality and limiting noise due to time delay between the measurement of the dependent and independent variables.

### Number of elderly residents

The number of residents that is 65 years or older is obtained from CBS register data for each zipcode-year combination from 2006 to 2018. Before 2014, this information is only available on a lower, 6-character zipcode level. For these years, we aggregate the information to four-digit zipcodes. In addition, some years record a fraction of inhabitants, or distinguish additional age groups. We recoded these records to the number of inhabitants that is 65 years or older. For the cross-sectional analysis, we select the number of elderly inhabitants in 2014.

### Presence of other collectives in the municipality

Four-digit zipcodes are linked to the municipalities of which they are a part using a linkage file from Statistics Netherlands. A binary variable is created, taking on a value of unity for each zipcode that is in a municipality containing at least one other zipcode with a care collective. In the longitudinal model, this is included as a time-constant variable (based on the inventory of collectives in 2020), since we often do not have information about the year in which these other care collectives in a municipality were founded.

### Control variables

We control for various time-varying neighborhood characteristics that have been found to relate to neighborhood cohesion and may also influence the emergence of citizen collectives. These are the number of inhabitants in a zipcode area [59], the fraction of inhabitants with a non-western migration background, the fraction of inhabitants with an income in the highest 20% of the Dutch income distribution [60] and the fraction of houses that is inhabited by home-owners [61]. All control variables are obtained from annual Statistics Netherlands public register data. For the cross-sectional analysis, we select the values from 2014.

### Missing data handling

There are some zipcode areas that correspond exactly to a neighborhood or city district, as defined by Statistics Netherlands. For some of these zipcodes, register data about the number of elderly residents and various control variables is missing, while the information *is* available in the corresponding neighborhood or district database. For these zipcode-year combinations, we impute the zipcode data from the neighborhood or district information. In total, we impute values for 17,689 zipcode-year combinations through this method.

This replacement results in 29,203 complete zipcode-year records and 20,887 remaining records with at least one missing value. These final missing values, which are almost exclusively on the control variables, are multiply imputed (m = 25) using predictive mean matching and classification and regression trees with the “mice” R package [62].

### Modelling

We expect that social cohesion in a neighborhood facilitates collective action of citizens, which aids in the emergence of care collectives. However, once a care collective has been established in a neighborhood, this potentially has a positive effect on social cohesion as well. Neighbors may have more contact through the collective and may feel more attached to the neighborhood when they are working together towards common goals. Thus, it is important to isolate the effect of cohesion on the emergence of citizen initiatives from the reversely causal effect. One way to do this is to analyze the data longitudinally. Unfortunately, data on the year in which a collective was founded is only available for 196 zipcode areas, out of a total of 896 in which a collective was known to be present in 2021. Therefore, to utilize all available information, we conduct two separate analyses. First, we estimate a cross-sectional multilevel logistic regression model using data on all the care collectives in our inventory. Next, we perform a discrete-time event history analysis (*basic frailty model*) using the available time-varying data. For both models, the levels of analysis are neighborhoods, nested in municipalities, nested in provinces.

For the cross-sectional multilevel logistic regression model (Table 1), we include 3831 Dutch zipcode areas (after excluding ambiguous zipcode areas that have fused or disbanded in the past years and zipcodes with fewer than 50 inhabitants). For the multilevel discrete-time event history analysis (Table 2), we also exclude zipcodes with fewer than 50 inhabitants, as well as all zipcodes from the model for which a care collective is recorded in our 2021 inventory, but we do not know the year in which it was founded. That leaves 3157 zipcodes (41042 observations). We use a Gompertz link function for the hazard and fit a series of nested models, similar to the cross-sectional analyses. In summary, we estimate 4 nested cross-sectional models, and 4 nested longitudinal models, the setup of which is briefly described below.

- First, we estimate an intercept-only model to partition the variance among the zipcode, municipal and province level.
- Second, we estimate a model with only the main independent variables, neighborhood contact and neighborhood attachment.
- Third, we add the control variables to the model at the zipcode and municipal level.
- Fourth, we estimate the interactions with our indicator for the necessity for care services (i.e. the number of elderly residents in the zipcode area).
- Fifth, we estimate the cross-level interactions with the presence of additional care collectives in the same municipality.

For the longitudinal models, we take exactly the same steps, except we include the effect of time (coded in years 2006–2018) at each step in the modelling process.

**Table 1:** Descriptive statistics for the dependent and independent variables of the cross-sectional analysis (*N* = 3831)

Variable	Mean	S.D.	Min	Max
Care collective	0.221	-	0	1
Contact	0.002	0.018	-	-
Attachment	0.001	0.015	-	-
Inhabitants > 65 years	787.534	736.501	0	5160
Inhabitants	4413.070	4172.149	50	28,410
Fraction non-western migrants	7.066	11.639	0	90
Fraction high incomes	22.384	9.295	0	0.724
Fraction house owners	66.344	17.422	0	1
Other initiatives	0.785	-	0	1

**Table 2:** Descriptive statistics for the dependent and independent variables of the longitudinal analysis (*N* = 41042)

Variable	Mean	S.D.	Min	Max
Care collective	0.058	-	0	1
Contact	0.002	0.017	-	-
Attachment	0.001	0.014	-	-
Inhabitants > 65 years	685.660	728.870	0	5347
Inhabitants	4109.930	4100.849	50	28,475
Other initiatives	0.740	-	0	1

## Results

### Descriptive results

**Fig. 1:**
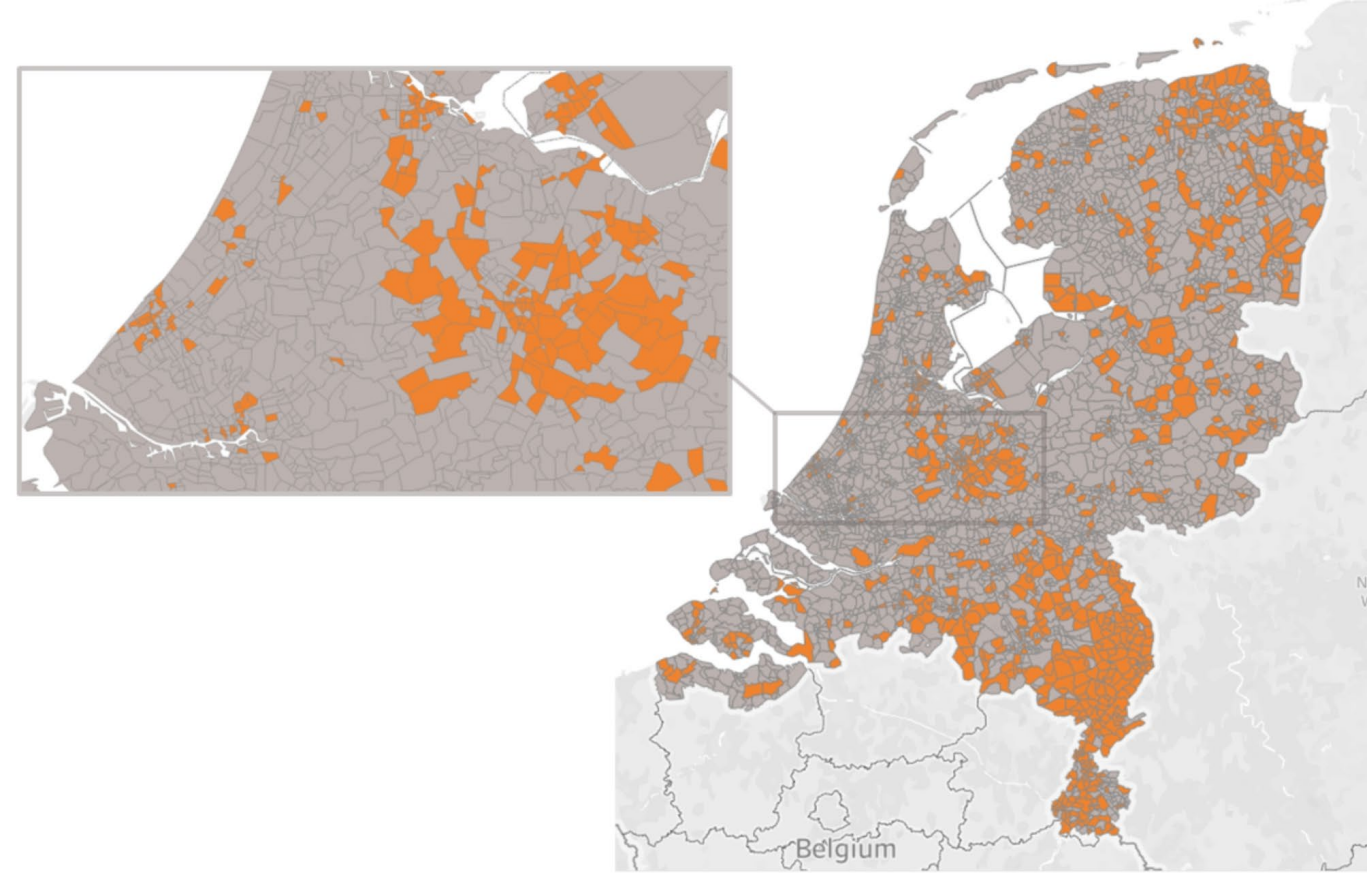
Overview of zipcode areas with at least 1 recorded initiative (in orange) Source: Own production created with ‘Tableau’ software

Figure 1 shows the zipcode areas of the Netherlands in which a care collective has been established (~ 20% of all zipcodes). It appears that care collectives are mostly clustered together, with concentrations in the middle, north-east and south-east of the Netherlands, as well as in and around some of the major cities. Coverage by collectives seems particularly widespread in the northern part of the Limburg (south-east) province. Upon further inspection of the data, this last concentration includes a large group of interlinked initiatives that provide shared dining for elderly residents. Because this is a distinct type of collective that is not extensively represented in the rest of the Netherlands, we perform a robustness check by separately analyzing a subset of the data that excludes zipcodes in which only collectives of this type are present. These results did not deviate from the main analyses.

With regard to feelings of neighborhood attachment and the prevalence of social relations in the neighborhood, variability is quite low between zipcodes. That is, these aspects of social cohesion did not vary strongly around the general average. To illustrate this further, the item-response models show that variability in answers to the survey questions between individuals, even after controlling for their personal traits, is around ten times larger than the variability that can be ascribed to the neighborhood.

### Cross-sectional analysis

Table 3 shows the results of the cross-sectional analyses of all 3831 available four-digit zipcode areas in The Netherlands. Model 1a shows an overall positive association between neighborhood attachment and the probability that a care collective is present in the neighborhood. We find no evidence for an association between neighborhood contact and the presence of a care collective. These findings persist when controlling for various potential confounders in Model 2a. Thus, we find support for our hypothesized association between neighborhood attachment and the presence of a citizen collective (H1), but not for an effect of the prevalence of social relations in the neighborhood (H2). In terms of effect size, the difference in the predicted probability (average marginal effect) of a care collective existing in a neighborhood that is one standard deviation below the mean level of attachment and one standard deviation above the mean is approximately 3% points.

Additionally, there seems to be a positive relation between the number of inhabitants in a neighborhood and the probability of a citizen collective. The fraction of non-western migrants and the number of high-income households are negatively related to the presence of a collective. Contrary to our expectation, we find no evidence that the number of elderly residents in the neighborhood is, on average, related to the emergence of a care collective (H3a), nor do we find evidence that the presence of other citizen initiatives in the same municipality is positively related to the presence of a citizen initiative in a neighborhood (H4a).

Model 3a, which includes the respective interaction terms of neighborhood attachment and neighborhood contact with the number of elderly residents, shows that the relation between neighborhood attachment and the probability of a care collective is stronger (more positive) if the number of elderly residents is larger. However, there is no evidence for an interaction between the number of elderly inhabitants and neighborhood contact. Thus, we find evidence in support of H3b, but not for H3c. More specifically, the results suggest that neighborhood attachment has no statistically distinguishable effect if the number of elderly residents is one standard deviation below the mean number of elderly inhabitants (b = -1.315, *p* = 0.472). However, when the number of elderly residents is one standard deviation above the mean, the relation turns positive and significant (b = 18.455, *p* = 0.001).

Finally, to test our expectation of a cross-level interaction between neighborhood attachment and contact with the presence of other care collectives in the municipality, we first test whether a model with random slopes for neighborhood attachment and contact yields significantly better fit. We find no support that this is the case. The LR-test is insignificant (*p* = 0.769), and the model yields higher AIC (Δ = + 1.4) and higher BIC (Δ = + 72.4) values than the model in which it is nested (Model 3). As a result, we conclude that there is no evidence that the effect of neighborhood attachment and contact varies between different municipalities, and therefore that there is no support for H4b and H4c. Nevertheless, the model including these interaction terms is included as Model 4 in the table, in which it can also be seen that both terms are insignificant. Very large standard errors in this model further indicate that the data do not support a complex model with random slopes well.

Overall, the intercept-only model reveals that variation in the presence of citizen collectives for care can be partially be attributed to variation at the level of municipalities (15%) and provinces (23%). Furthermore, pseudo-R^2^ values reveal that the best-fitting model is able to account for around 11% of the variation in the presence of citizen collectives for care. While this may not seem like a particularly high fraction of the variation, it is important to note that we only include some aggregate community-level factors in these models, and forego the dynamics between individual citizens.

**Table 3:** Multilevel (N_zipcode_=3831, N_municpality_=352, N_province_=12) logistic regression of the presence of a collective for care on the predictor variables. Number of elderly residents is centered on its grand mean

	Model 0a	Model 1a	Model 2a	Model 3a	Model 4a
Variable	**Coef. (s.e.)**	**Coef (s.e.)**	**Coef (s.e.)**	**Coef (s.e.)**	**Coef (s.e.)**
Intercept	-1.471*** (0.329)	-1.465*** (0.331)	-19.435 (409.893)	-10.531 (456.027)	-19.545 (439.681)
Contact		-3.779 (2.992)	-0.566 (3.025)	-0.033 (4.656)	4.358 (28333.590)
Attachment		8.941** (3.410)	9.596** (3.635)	8.570 (5.771)	-0.492 (38799.040)
Inhabitants > 65 years (x1000)			0.149 (0.129)	0.146 (0.131)	0.181 (0.132)
Inhabitants (x1000)			0.097*** (0.026)	0.096*** (0.026)	0.095*** (0.026)
Fraction non-western migrants			-0.019** (0.007)	-0.017* (0.007)	-0.017* (0.007)
Fraction high incomes			-0.022* (0.009)	-0.023** (0.009)	-0.021* (0.008)
Fraction house owners			0.003 (0.005)	0.004 (0.005)	0.004 (0.005)
Attachment * Inhabitants > 65 years				13.422** (5.123)	
Contact * Inhabitants > 65 years				-1.882 (4.192)	
Other initiatives			18.292 (409.893)	18.280 (456.026)	18.247 (439.681)
Attachment * Other initiatives					10.152 (38799.040)
Contact * Other initiatives					-4.209 (28333.690)
AIC	3386.5	3382.3	3063.2	3058.6	3059.2
BIC	3404.1	3414.0	3132.0	3141.2	3213.6
Log-likelihood	-1690.5	-1686.7	-1520.6	-1517.3	-1513.1
ICC (municipal)	0.152				
ICC (province)	0.228				
Pseudo-R^2^		0.02	0.10	0.11	0.11

### Longitudinal analysis

Next, we turn to the results of the temporal analyses, which includes richer time-varying data for a subset of the four-digit zipcode areas and the control variables. This helps us to identify potential issues of reverse causality and reduces noise due to time differences between the measurement of the independent and dependent variable. The results of these analyses are summarized in Table 4.

Model 0b shows that the hazard rate increases with time. The exponentiated coefficient (1.20) indicates that, with every year, a zipcode area is about 20% more likely to gain a citizen collective if one hasn’t emerged up to that moment.

Model 1b shows no evidence that either neighborhood attachment or neighborhood contact affect the probability that a care collective emerges in the neighborhood. This conclusion remains after inclusion of the control variables (Model 2b). Thus, contrary to our cross-sectional analyses, we find no support for H1 or H2 in our survival analysis. In addition, there also does not appear to be a statistically distinguishable effect of the number of inhabitants on the probability that a care collective emerges in a neighborhood. Model 2b shows no statistical evidence that the number of elderly residents or the presence of other care collectives in the municipality has an effect on the probability that a care collective emerges, and thus does not provide support for H3a and H4a. Model 3b shows no statistical support for our expectation that the effect of neighborhood attachment (H3b) and contact (H3c) is moderated by the number of elderly inhabitants. Finally, we find no evidence that the effect of neighborhood attachment and contact vary between municipalities (LR = 1.035, *p* > 0.999), and thus find no support for H4b and H4c. Again, the model’s insignificant cross-level interaction terms are recorded in the table for reference, though the data do not support the added complexity of random slopes in general, which is also reflected by the large inflation of the error terms in the model.

**Table 4:** Multilevel (N_zipcode−year_=41042, N_zipcode_=3157, N_municpality_=345, N_province_=12) survival analysis for the emergence of a collective for care. The number of elderly inhabitants is centered on its grand mean

	Model 0b	Model 1b	Model 2b	Model 3b	Model 4b
Variable	**Coef. (s.e.)**	**Coef (s.e.)**	**Coef (s.e.)**	**Coef (s.e.)**	**Coef (s.e.)**
Intercept	-371.836*** (43.160)	-372.376 (1860.045)	-344.216 (1041.393)	-358.021 (1267.600)	-362.602 (1348.200)
Year	0.182*** (0.021)	0.182*** (0.005)	0.168*** (0.019)	0.166*** (0.014)	0.168*** (0.012)
Contact		2.135 (4.019)	5.467 (5.234)	6.811 (7.881)	-2.659 (97384.402)
Attachment		1.362 (3.192)	2.152 (5.806)	-2.112 (10.070)	1.530 (12390.490)
Inhabitants > 65 years (x1000)			0.189 (0.197)	0.271 (0.204)	0.178 (0.171)
Inhabitants (x1000)			0.043 (0.039)	0.032 (0.041)	0.051 (0.033)
Attachment * Inhabitants > 65 years				-3.083 (9.031)	
Contact * Inhabitants > 65 years				6.022 (6.833)	
Other initiatives			17.258 (425.042)	18.201 (1173.030)	18.430 (13481.402)
Attachment * Other initiatives					-8.944 (129305.100)
Contact * Other initiatives					11.220 (973803.900)
AIC	2363.0	2366.9	2353.4	2274.1	2278.4
BIC	2406.3	2426.6	2428.2	2382.5	2383.2
Log-likelihood	-1176.0	-1176.9	-1166.6	-1126.6	-1127.2
ICC (zipcode)	0.001				
ICC (municipal)	0.186				
ICC (province)	0.157				
Pseudo-R^2^		0.00	0.01	0.04	0.04

In summary, we find cross-sectional associations between neighborhood attachment and the probability that a care collective emerges in a neighborhood. We find that this association is stronger if there are more elderly residents in the neighborhood. However, we cannot find these associations in the subset of the data where we can ensure temporal precedence.

### Robustness

A number of decisions have been made in the operationalization and analytical strategy that may have affected these results. Robustness of the cross-sectional findings is investigated by varying the year that was used to determine the values of the independent and control variables between 2012 and 2015. Robustness of the longitudinal models is investigated by performing the analyses on a subset of the data that included only those zipcode areas for which no other care collectives were present than the ones for which we knew the founding year. Furthermore, polynomial specifications for the effect of time and different link functions for the hazard rate were considered. For both cross-sectional and longitudinal models, we vary the items that were included in the ecometric approach to account for unintended composition effects, we vary the magnitude of the shrinkage factor in the ecometric design, and we vary the subset of zipcode areas to exclude areas with large concentrations of care collectives that were mainly oriented towards shared dining. Finally, we specified our models with quadratic and cubic terms of our main independent variables: neighborhood contact and attachment. We found no support from these models that support curve-linear effects. Overall, each of our alternative specifications yielded qualitatively identical interpretations, with narrow intervals of deviation around the main analyses’ point estimates.

## Discussion

In this study, we provided one of the first large-scale empirical accounts of the emergence of citizen care collectives. We thereby focused on community-level explanations, and tested hypotheses that social cohesion, necessity for care services, and models for collective action (knowledge obtained from other collectives in the same area) could facilitate collective action by citizens in the care domain. We differentiated between two aspects of social cohesion: feelings of attachment and belonging and the prevalence of social contacts in the neighborhood, which we measured using the ecometric approach [54]. We tested our hypotheses using a comprehensive inventory of Dutch citizen care collectives, which we enriched with survey data on social cohesion and neighborhood (which we defined as 4-digit zipcode areas) characteristics from public register data.

Our main conclusion is that there is a positive association between the extent to which inhabitants experience feeling of attachment and belonging to a neighborhood and the presence of a care collective in that neighborhood. A care collective is more likely to exist in a neighborhood when its inhabitants have, on average, stronger feelings of attachment and belonging to that neighborhood. This is in line with our theoretical reasoning that neighborhood attachment can facilitate collective action by fostering a shared social identity, which motivates inhabitants to pursue goals that benefit their community. It is also consistent with literature on other forms of collective action, in which collective mobilization of citizens has been linked to social identity and group efficacy [63].

Moreover, we found that attachment has a stronger association with the emergence of collective action when there is sufficient necessity for a collective’s care services, as measured by the number of elderly residents in a neighborhood. This is in line with our proposed extension of previous literature, which has argued for direct relations between collective action, necessity, and social cohesion [15, 17], but did not yet consider their mutual reinforcement. However, we did not find that the necessity for services had a direct association with the emergence of care collectives in this study. This is in contrast to previous literature, which conducted in-depth interviews with existing collectives and found that the presence of elderly residents was an important factor for the emergence of collectives [17]. However, our study shows that this does not translate straightforwardly to an observable empirical pattern for the entirety of the Netherlands. Potentially, the direct effect of necessity in previous research was caused by survivor bias of the sample, and instead, the effect of necessity for care services is conditional on the fulfillment of other predictors of collective action, such as neighborhood attachment. Thus, while inductive research has shown the potential importance of elderly residents in establishing collectives, the lack of a general association in our study shows that the conditions and mechanisms through which the presence of elderly residents (and by assumption the necessity for care services) can foster the emergence of citizen collectives are not yet sufficiently understood.

Taken together, we conclude that there is variability in the ability of neighborhoods to organize collective care services. The necessity for a care collective’s services is not by itself a sufficient condition for the emergence of collective action, and areas with low levels of community attachment may be particularly vulnerable to a retreat of care institutions in their area.

Our second conclusion is that we found no evidence that the prevalence of social relations in a neighborhood is related to the emergence of care collectives. This is inconsistent with our theoretical expectation that citizens would be more likely to participate in care collectives if their neighborhoods are characterized by many social relations, which increases the probability of future gains from said collective through trust and reciprocity [40, 44, 64]. These findings deviate from literature on other forms of citizen collective action, such as mutual insurance, where group-level and individual prevalence of social relations were shown to foster collective action through increased trust and commitment [8]. Similarly, research on energy cooperatives in the Netherlands showed that these initiatives were more likely to exist in municipalities with high density of social relations [65]. This may suggest that collective health care provision deviates from other collectives in terms of the required conditions for its emergence. There may be a more important role for social identity and resulting solidarity mechanisms in health care collectives than citizen collectives in other domains.

Thirdly, we find no evidence that the presence of other care collectives in the municipality is related to the emergence of a new collective, nor do we find evidence that the association between attachment and contact and the emergence of a citizen collective varies between municipalities in general. This conclusion deviates from previous research, which found that geographic distance to the nearest zipcode with a collective was predictive for the emergence of a care collective [15]. While citizen collectives for care do appear to be geographically clustered, we do not find that this is particularly salient within municipal boundaries. Our understanding of the mechanisms that drive this clustering remains incomplete, particularly regarding the role of legislative boundaries in the interplay between existing and emerging citizen collectives for care. Further study is required to understand if geographic patterns in the emergence of citizen collectives can be attributed to mechanisms such as the diffusion of models for collective action and inherited legitimacy of collectives.

Importantly, limitations in the availability of data in our study make that we remain reserved regarding the causality of the relation between neighborhood attachment and care collectives. We did not find an association between attachment and care collectives for the sample of the data that ensured temporal precedence. This may imply an issue of reverse causality, in which the presence of citizen collectives for care strengthens feelings of attachment to the neighborhood. However, a longitudinal model in which attachment was regressed on citizen collective presence also did not yield any significant association, and therefore does not provide support for an argument of reverse causality. In addition, the findings of the cross-sectional model were robust to the selection of earlier years for the measurement of neighborhood attachment, and there was little within-neighborhood variation in levels of attachment over the years. It may also be that the divergent conclusion from the longitudinal model is explained by the sample on which it was fitted, which was based on convenience and considerably smaller than the complete inventory of collectives. Thus, while we concluded that we find evidence for an association between neighborhood attachment and the emergence of care collectives, we cannot confidently assess the causality of this relation yet.

In addition, it is possible that the absence of a relationship between neighborhood contact and the emergence of care collectives is because contact measured at the neighborhood level is too coarse to capture the intricacies of contact networks among neighbors that facilitate trust and processes of mobilization that have been reported in qualitative literature to date [22]. Moreover, since neighborhood attachment and contact were highly correlated, it is also possible that contact plays a role in a more complex interplay with neighborhood attachment, the investigation of which falls outside of the scope of the current study. And more generally, there may be other conditionalities that govern the mechanisms that were central to our study, such as differences between urban and rural settings for example. To address these issues, further research would benefit from mapping the network topology of a neighborhood and combining it with information about participation in care collectives. This would allow for a more direct test of hypotheses regarding contagion/invitation processes between citizens and the relation between trust formation in the neighborhood and collective action.

In terms of the scope of the study, our data confined us to a single national context. The Netherlands is among the first countries to exhibit such widespread and coordinated emergence of citizen collectives for care, and we are currently unable to compare our results across countries with different legislation and social policies with respect to citizen-based initiatives. Nevertheless, a trend towards citizen-based organization of (care) services is common to many (Western) societies [66], and we believe that the insights are informative across national contexts. This is because the most impactful legislation that influences citizen collectives is situated at the municipal level, to which only around 15% of variation in the existence of care collectives could be attributed in our analyses. Next to this, the theoretical framework from which we derive our hypotheses is based on international research into social exchange and neighborhood identity. Thus, while national social policy likely plays a role in the establishment of citizen collectives for care, and some level of institutional support may be required to see citizen collectives emerge, we expect that the mechanisms of neighborhood identity and necessity for care will also be important in other contexts.

Finally, our inventory of care collectives may be incomplete. There may be selectivity favoring the inclusion of larger care collectives, as well as collectives that are more actively involved in collaborations with municipalities or other organizations. However, the inventory by NLZVE is one of the most extensive efforts to map the presence of Dutch care collectives to date, and it allows us to study the emergence of citizen collectives at a large scale, linking it to administrative data within the Netherlands.

## Conclusion

We have shown that the study of citizen care collectives can benefit from the inclusion of explanations at the community-level. Using a novel and extensive inventory of health care collectives in the Netherlands, we have shown that the presence of collectives for care is associated with feelings of neighborhood attachment, and that this association is stronger if there is more demand for a collective’s services. Simultaneously, we did not find empirical support for other mechanisms that were implied by previous research. The quality, transparency and availability of data is still developing in the field of citizen collectives, and are in some cases still too coarse to capture mechanisms of interest unequivocally. To further understand the extent to which the discrepancies between previous qualitative work and the current study are driven by theoretical incompleteness (e.g. specification of the conditions and contexts in which cohesion and necessity facilitate collective action) or by methodological artefacts (e.g. sampling biases or levels of measurement), we would like to emphasize the opportunity for future research to collect and combine data at the micro level and the macro level. That is, to obtain information about which specific inhabitants of a region are members of a citizen collective for care, so that their individual characteristics, as well as those of the community in which they are embedded, can be brought together into one model for the emergence of collective action. We have shown that neighborhood identity may be vital element of such a model.

## Electronic supplementary material

Below is the link to the electronic supplementary material.

Supplementary Material 1

## Data Availability

The datasets generated and/or analysed during the current study are not publicly available due to restrictions imposed by Statistics Netherlands and Nederland Zorgt voor Elkaar on their usage, but are available from the corresponding author on reasonable request.

## Abbreviations

NLZVE: Nederland Zorgt Voor Elkaar
