# Supplementary material for: The emergence of citizen collectives for care: the role of social cohesion
Source: BMC Public Health. 2024 Dec 2;24:3360. doi: 10.1186/s12889-024-20780-7 (PMC11613483; doi:10.1186/s12889-024-20780-7)
Supplement: Supplementary file 1 — Supplementary Material 1 [file 12889_2024_20780_MOESM1_ESM.docx]

**Appendix A – WoON survey items**

Items used for the measurement of attachment and contact in the ecometric approach (translated from original language: Dutch).

Neighborhood contact:

Intro question: Please indicate to what extent you agree with the following statements about your neighborhood.

| Item name | Statement | Answer categories |
| --- | --- | --- |
| ConBuur1 | I have a lot of contact with my direct neighbors | 1 – Totally agree  2 – Agree  3 – Neither agree nor disagree  4 - Disagree  5 - Totally disagree |
| ConBuur2 | I have a lot of contact with other inhabitants of the neighborhood | 1 – Totally agree  2 – Agree  3 – Neither agree nor disagree  4 - Disagree  5 - Totally disagree |
| MensKen | People in this neighborhood hardly know each other | 1 – Totally agree  2 – Agree  3 – Neither agree nor disagree  4 - Disagree  5 - Totally disagree |

Neighborhood attachment:

Intro question: Hereafter follow a few statements about the neighborhood in which you live. Please indicate for each statement to what extent you agree or disagree with the statement.

| Item name | Statement | Answer categories |
| --- | --- | --- |
| TGehecht | I feel attached to this neighborhood | 1 – Totally agree  2 – Agree  3 – Neither agree nor disagree  4 - Disagree  5 - Totally disagree |
| Brtthuis | I feel at home in this neighborhood | 1 – Totally agree  2 – Agree  3 – Neither agree nor disagree  4 - Disagree  5 - Totally disagree |

**Appendix B – Overview of ecometric approach**

The benefits of the ecometric approach are obtained by fitting a multi-level item response model for each of the two dimensions of social capital, where the respective items are nested in respondents, which are nested in neighborhoods. This comes down to estimating the following equation for both dimensions of social cohesion (Equation 1).

$$Y_{ijk}=\gamma_{000}+\sum_{m=1}^{M} \alpha_{m}D_{mijk}+\sum_{q=1}^{Q} \delta_{q}X_{qjk}+\upsilon_{00k}+u_{0jk}+e_{ijk}$$

Where $Y_{ijk}$ is the response to item *i* by person *j* in neighborhood *k*, $\gamma_{000}$ is the grand mean of the items, $D_{mijk}$ are *M - 1* dummy variables for each of the items (1 serves as reference), $X_{qjk}$ are the subject-level variables and $\upsilon_{00k}$, $u_{0jk}$ and $e_{ijk}$ are the residuals at the neighborhood, subject and item level respectively. Of particular interest are the neighborhood residuals $\upsilon_{00k}$ these are the deviations from the grand mean of social cohesion that cannot be attributed to respondent characteristics. Positive values indicate that social cohesion is higher than average in a neighborhood, and negative values indicate that cohesion it is lower than average. The neighborhood residuals $\upsilon_{00k}$ are calculated by multiplying the raw average residuals per neighborhood $r_{j}$ by a shrinkage factor. This shrinks neighborhood deviations to the general average depending on the subject-level variation $\sigma_{j}$ and number of respondents per neighborhood $N_{k}$ (Rasbash et al., 2009). These neighborhood residuals subsequently serve as independent variables in our analysis (Equation 2).

$$\upsilon_{00k}=r_{k}\frac{\sigma_{k}}{\sigma_{k}+\frac{\sigma_{j}}{N_{k}}}$$

This results in the following scales:

| Year | Mean (s.d.) attachment | Mean (s.d.) contact | $\mathbf{N}_{\boldsymbol{neighborhood}}$ | $\mathbf{N}_{\boldsymbol{respondent}}$ |
| --- | --- | --- | --- | --- |
| 2006 | 0.001 (0.020) | 0.002 (0.020) | 3422 | 64000 |
| 2009 | <0.001 (0.015) | 0.003 (0.020) | 3312 | 68831 |
| 2012 | <0.001 (0.015) | 0.003 (0.021) | 3364 | 60044 |
| 2015 | <0.001 (0.015) | 0.002 (0.019) | 3392 | 55223 |
| 2018 | <0.001 (0.015) | 0.001 (0.017) | 3408 | 58849 |
